# Changes in telomere length in Arabidopsis thaliana plants infected with the phytopathogenic bacteria Pseudomonas syringae DC3000

**DOI:** 10.18699/vjgb-26-73

**Published:** 2026-09

**Authors:** A.V. Sannikova, L.R. Abdulkina, M.R. Sharipova, L.R. Valeeva

**Affiliations:** Institute of Fundamental Medicine and Biology, Kazan (Volga Region) Federal University, Kazan, Russia; Institute of Fundamental Medicine and Biology, Kazan (Volga Region) Federal University, Kazan, Russia; Institute of Fundamental Medicine and Biology, Kazan (Volga Region) Federal University, Kazan, Russia; Institute of Fundamental Medicine and Biology, Kazan (Volga Region) Federal University, Kazan, Russia

**Keywords:** plant telomeres, DNA stability, biotic stress, bacterial infection, ribosomal proteins, rRNA methyltransferase, phytopathogens, теломеры растений, стабильность ДНК, биотический стресс, бактериальная инфекция, рибосомные белки, метилтрансфераза рРНК, фитопатогены

## Abstract

Telomeres are specialized nucleoprotein complexes at the physical ends of linear chromosomes of most eukaryotes that represent a key structural element in genome stability regulation. One of the most important characteristics determining the protective capacity of telomeres is telomere length (TL). In Arabidopsis thaliana, telomere length varies among ecotypes, which may be considered as an adaptive mechanism to various environmental and stress conditions, including phytopathogenic infections. However, the mechanisms of TL regulation under biotic stress remain unclear. Here, we show the results of our study of the TL changes in A. thaliana plants under the Pseudomonas syringae pv. tomato DC3000 infection. In the study, we used natural ecotypes Col-0 (medium telomere length) and Sf-2 (long telomeres), as well as mutant plant lines with disordered and wild-type telomere length phenotypes, oli5- 2–/– and nop2c-2–/–, respectively. It was found that susceptibility to the phytopathogen varies in plants: the line with short telomeres, oli5-2–/–, showed the highest sensitivity compared to the WT and nop2c-2–/–. Sf-2 plants possessed the highest resistance to the infection. Significant shortening of TL on the third day after infection was found on the short arm of chromosome 4 of infected nop2c-2–/– plants, whereas TL of infected oli5-2–/– plants slightly decreased on the third day and increased on the seventh day after infection. The most resistant line Sf-2 retained TL on the third and seventh days after infection but significantly lost it on the 18th day. So, it was shown for the first time that phytopathogenic infection affects telomere length in plants, which opens a new direction in the study of plant telomere regulation under biotic stress.

## Abstract (ru)

Ключевым структурно-функциональным элементом, обеспечивающим стабильность генома большинства эукариот, являются теломеры – специализированные нуклеопротеиновые комплексы на физических концах линейных хромосом. Одна из важнейших характеристик теломер, определяющих их защитный потенциал, – длина теломерной ДНК. У растений Arabidopsis thaliana она варьирует среди экотипов, что может указывать на роль теломер в адаптации к различным условиям окружающей среды и стрессу, включая воздействие фитопатогенов. Однако механизмы регуляции длины теломер при биотическом стрессе остаются неизученными. В статье представлены результаты исследования зависимости длины теломер A. thaliana от инфицирования фитопатогенным штаммом Pseudomonas syringae pv. tomato DC3000. В работе использовались природные экотипы растений Col-0 (средняя длина теломер) и Sf-2 (длинные теломеры), а также мутантные линии с нарушением регуляции длины теломер и с теломерным фенотипом дикого типа, oli5-2–/– и nop2c-2–/– соответственно. Установлено, что восприимчивость к фитопатогену различается у растений разных генотипов: линия oli5-2–/– с короткими теломерами проявляла наибольшую чувствительность, в том числе по сравнению с диким типом и линией nop2c-2–/–, в то время как растения экотипа с длинными теломерами Sf-2 отличались наибольшей устойчивостью. Значимое укорочение длины теломер на третьи сутки после инфицирования было обнаружено на коротком плече хромосомы 4 у инфицированных растений линии nop2c-2–/–, тогда как длина теломер инфицированных растений oli5-2–/– незначительно сокращалась на третьи сутки и возрастала на седьмые сутки после инфицирования, что указывает на динамичность регуляции длины теломер при стрессе. Длина теломер растений дикого типа значительно не изменялась при инфицировании. Наиболее устойчивая линия Sf-2 сохраняла длину теломер на третьи и седьмые сутки после инфицирования, но на 18-е сутки происходило значительное укорочение теломер. Таким образом, впервые показано, что фитопатогенная инфекция влияет на длину теломер у растений, что открывает новое направление в изучении их регуляции при биотическом стрессе.

## Introduction

The interaction of living organisms with the environment requires diverse and finely regulated adaptive mechanisms to maintain homeostasis, among which genome protection plays a crucial role. Biotic stress can enforce enormous pressure on the maintenance of genome integrity. In animal cells, microbial metabolites such as colibactin E have been shown to exert genotoxic effects on host DNA, including through alkylation, leading to its degradation (Wilson et al., 2019; Xue et al., 2019; Puschhof, Sears, 2022). Furthermore, pathogens such as Helicobacter pylori and Staphylococcus aureus can induce double-strand breaks and fragmentation of human DNA (Chakraborty et al., 2011; Toller et al., 2011). Biotic stress, particularly that induced by phytopathogenic microorganisms, also causes complex and multilevel effects on plant organisms (Kong, Yang, 2023; Solomon et al., 2024). At the genomic level, microbial infections can induce double-strand DNA breaks in plants, alter chromatin structure and architecture, and trigger changes in DNA and histone methylation as part of the stress response (Pavet et al., 2006; Dowen et al., 2012; López Sánchez et al., 2016; Alonso et al., 2018; Hannan Parker et al., 2022; Corrêa et al., 2024).

In turn, telomeres are among the fundamental structural and functional elements responsible for maintaining chromatin integrity in plants, as in most other eukaryotes (McKnight et al., 2002; Riha, Shippen, 2003; Zakian, 2012; Peska, Garcia, 2020; Shakirov et al., 2022). Telomeres are specialized nucleoprotein structures located at the ends of the linear chromosomes of most eukaryotes that consist of tandem DNA repeats together with associated protein complexes. They maintain linear genome stability by preventing chromosome ends from being recognized by endogenous exonucleases and DNA repair machinery and by avoiding pathological chromosome fusions (Riha, Shippen, 2003). In addition, telomeres are involved in controlling unprogrammed genome degradation during cell division, which contributes to replicative senescence (Watson, Riha, 2010). Therefore, telomere shortening is considered an important marker of genome instability (Gan, 2003; Amiard et al., 2014; Shakirov et al., 2022; Agabekian et al., 2024; Di Pietro et al., 2024). Stress can also affect telomere structure, promote telomeric DNA shortening, and reduce telomerase activity. However, the relationship between telomere regulation and infectious diseases under both laboratory and natural conditions remains poorly understood, and the underlying mechanisms remain controversial (Karell et al., 2017; Giraudeau et al., 2019; Sudyka et al., 2019; Wolf et al., 2023; Ravindran et al., 2024). Moreover, reliable information regarding the effects of biotic stress on the regulation of telomere length in plants is currently lacking.

Telomere length in A. thaliana is known to vary widely among hundreds of characterized ecotypes (Riha et al., 2002; Shakirov, Shippen, 2004; Abdulkina et al., 2019; Choi et al., 2021). Moreover, it has previously been shown that the molecular mechanisms regulating telomeres are closely linked to the maintenance of the structure and function of another important subcellular structure – the ribosome (Abdulkina et al., 2019; Valeeva et al., 2023). Accordingly, several proteins associated with ribosome biogenesis have been identified as positive regulators of telomere length in Arabidopsis (Abdulkina et al., 2019; Agabekian et al., 2024). The NOP2A/ OLI2 gene of A. thaliana encodes a key enzyme involved in 25S rRNA methylation and maturation of the 60S ribosomal subunit, and mutation of this gene is followed by severe defects in plant development and metabolism (Maekawa, Yanagisawa, 2021), as well as by a 27 % reduction in telomere length relative to wild-type plants (Abdulkina et al., 2019). The NOP2A paralog, NOP2C/NSUN5, also belongs to the NOP2/NSUN family of 25S rRNA methyltransferases (Burgess et al., 2015). However, mutations in the gene encoding this enzyme do not lead to changes in telomere length (Kobayashi, 2018), indicating functional divergence between these paralogs. Telomere shortening in A. thaliana was also observed in mutants of the ribosomal protein L5 paralog genes OLI5/RPL5A and OLI7/ RPL5B (Abdulkina et al., 2019). In addition, more data indicate that ribosomal proteins, as well as ribosome-binding and ribosome-modifying proteins, play an important role in stress adaptation (Schosserer et al., 2015; Tieu Ngoc et al., 2021; Fakih et al., 2023; Siodmak et al., 2023; Wang et al., 2024; Cai et al., 2025; Fakih, Germain, 2025).

In this work, we studied the effect of bacterial infection on telomere length in A. thaliana. In our study, we used plant lines differing both in genotype and in telomere length, including two natural A. thaliana Col-0 and Sf-2 ecotypes, characterized by intermediate and long telomeres, respectively, as well as two mutant lines: oli5-2–/–, with short telomeres, and nop2c-2–/–, with intact telomere length. Therefore, our findings represent an important first step toward understanding the regulation of telomere length in plants under biotic stress.

## Materials and methods

Plant material and bacterial strains. Arabidopsis thaliana ecotypes Columbia-0 (Col-0) and San Feliu-2 (Sf-2), as well as the mutant lines nop2c-2–/– (At1g06560; encoding the rRNA S-adenosyl-L-methionine-dependent methyltransferase NOP2C) and oli5-2–/– (At3g25520; encoding the large ribosomal subunit protein OLI5/RPL5A), were used in this study. Second-generation (G2) mutant plants were homozygous for the T-DNA insertion in the target gene. Seeds were obtained from the Arabidopsis Biological Resource Center (ABRC; Ohio State University, USA).

Seeds were surface-sterilized in sodium hypochlorite solution according to a standard protocol (Lindsey et al., 2017). After sterilization, seeds were stratified at 4 °C for 24 h. Subsequently, 10 seeds were plated on solid MS medium (Murashige and Skoog basal medium: 1/2 MS, 1 % sucrose, pH 5.6) in Petri dishes and grown for 14 days under standard conditions (16 h light/8 h dark photoperiod, 20–22 °C, 45–60 % relative humidity, light intensity 7,000–9,000 lux) in a growth chamber (Sanyo, Japan). The phytopathogenic strain Pseudomonas syringae pv. tomato DC3000 (Pst DC3000) from the laboratory collection was used for plant infection experiments. Pst DC3000 cultures were grown at 28 °C in liquid MG medium (mannitol-glutamate medium; mannitol, 54.9 mM; glutamic acid, 6.8 mM; KH2PO4, 0.735 mM; CaCl2, 1.80 mM; NaCl, 3.42 mM; MgSO4·7H2O, 0.812 mM; FeSO4, 1.78 mM; Na- EDTA, 0.994 mM; pH 7.0) supplemented with rifampicin (50 μg/mL) under shaking at 250 rpm.

Plant infection was performed using an optimized floodinoculation method developed for A. thaliana seedlings grown in Petri dishes (Ishiga et al., 2011). Prior to infection, all plant lines were cultivated for 14 days under the standard conditions described in section “Plant material and bacterial strains”. Pst DC3000 cultures were grown for 12–14 h to an optical density of OD600 = 0.4, also as described above. Before plant inoculation, bacterial cells were harvested by centrifugation at 5,000 rpm, washed twice to remove residual medium, and resuspended in 2.5 % sucrose solution supplemented with 0.025 % Silwet L-77. Control plants were treated with sterile 2.5 % sucrose solution containing 0.025 % Silwet L-77 without bacteria. Plant stress responses and visible disease symptoms were monitored and photographed at 1, 3, 5, and 7 days post infection (dpi); plants of the Sf-2 line were additionally monitored for up to 18 dpi. For each plant line, experiments were performed in at least three biological replicates, including three control groups and three infected groups for each time point. Plant tissue samples for telomere length analysis were collected at 3, 5, and 7 dpi, as well as at an additional 18 dpi for the Sf-2 line.

Analysis of bacterial infection in plant tissues. To evaluate bacterial colonization of A. thaliana tissues by P. syringae DC3000, bacteria were reinoculated from both the plant surface and homogenized plant tissues as described by Y. Ishiga et al. (2011). The analysis was conducted at six days post infection.

Analysis of plant telomere length. Genomic DNA for telomere length analysis was isolated from plant tissues using a standard CTAB-based extraction method (Castillo-González et al., 2022). Telomere lengths at specific chromosome arms were analyzed using the PETRA (Primer Extension Telomere Repeat Amplification) assay with primers specific to the subtelomeric regions of chromosome arms 4 and 5: primer 4R for the short arm of chromosome 4, and primers 5L and 5R for the short and long arms of chromosome 5, respectively (Mayer et al., 1999; Tabata et al., 2000; Heacock et al., 2004; Watson, Shippen, 2007). PCR products were analyzed by non-radioactive Southern blot hybridization using a DIG-labeled telomeric DNA probe (Sigma-Aldrich, USA) and DIG-specific antibodies (Roche, USA). Mean telomere lengths were calculated using the WALTER online tool (https://www.ceitec.eu/chromatinmolecular- complexes/rg51/tab?tabId=125#WALTER) (Lyčka et al., 2021).

Statistical analyses were performed using GraphPad Prism version 8.0 (GraphPad Software, USA). Mean telomere length values and standard deviations were used for data visualization and graph generation. Statistical significance was assessed using a two-sided t-test, and differences were considered statistically significant at p < 0.05.

## Results

**Differences in susceptibility to phytopathogenic infection among plants with different genotypes**

To identify an association between the biotic stress responses and telomere length regulation, 14-day-old A. thaliana plants of four genotypes were infected with the phytopathogenic strain P. syringae DC3000 (Supplementary Fig. S1)1:

Supplementary Materials are available in the online version of the paper: https://vavilov.elpub.ru/jour/manager/files/Suppl_San_Engl_30_5.pdf


1) the control ecotype Col-0, with a telomere length of 2.0–5.0 kb (Shakirov, Shippen, 2004); 2) the Sf-2 ecotype, with long telomeres of 4.5–7.0 kb (Abdulkina et al., 2019); 3) the oli5-2–/– mutant line, deficient in the ribosomal protein gene OLI5/RPL5A (telomeres are shorter compared to the Col-0 wild type, 1.8–2.0 kb); 4) the nop2c-2–/– mutant line, deficient in the gene NOP2C of the S-acetyl-L-methionine-dependent rRNA methyltransferase, a regulator of ribosome biogenesis (the average telomere length across all chromosomes remains intact compared to the Col-0 wild type) (Kobayashi, 2018).

To confirm infection and the invasion of the phytopathogen into plant cells, we performed bacterial plating both from surface washes and from homogenates derived from surfacesterilized tissues of control and infected plants. It was found that the phytopathogenic strain is present within both the intracellular and intercellular tissue compartments, thereby confirming the pathogen’s invasion into the intercellular spaces of the tissues as well as into the interior of the plant cells (Fig. S2). The presence of Pst DC3000 cells was also detected on the plant surfaces (Fig. S2). In the control group (uninfected plants), no colonies of P. syringae DC3000 were detected, neither on the surface nor within the plants, indicating an absence of contamination during the experiment (Fig. S2).

During the experiment, all plants in the uninfected control groups showed normal growth and no visible signs of stress (Fig. 1). In contrast, infected plants displayed distinct responses to the phytopathogen depending on the genotype. No noticeable morphological or phenotypic changes were observed in any of the infected plant lines during the first two days after infection. However, by three days post infection, symptoms of leaf pathology became evident, with the most pronounced effects observed in the mutant line oli5-2–/–. In oli5-2–/– plants, changes in leaf pigmentation were observed, indicating the accumulation of anthocyanins, protective pigments that are produced in response to various stress factors and contribute to protection against increased levels of reactive oxygen species (ROS) (Gould, 2004; Li, Ahammed, 2023). In addition, chlorosis was detected, primarily in the cotyledons. Chlorosis is a characteristic symptom of DC3000 infection in Arabidopsis (Katagiri et al., 2002) and is associated with the progression of leaf senescence. Symptoms of tissue damage, including chlorosis, necrosis, and leaf wilting, progressively increased in oli5-2–/– plants throughout the experiment. By seven dpi, tissue damage had reached a critical level, and the experiment was terminated for this line (Fig. 1). The nop2c-2–/– mutant and wild-type Col-0 plants exhibited higher resistance to infection. At three dpi, plants of both lines retained a darker green coloration, showed no obvious anthocyanin accumulation, and displayed fewer chlorotic leaves than oli5-2–/– plants. Disease symptoms became more pronounced at five dpi and reached severe levels by seven dpi (Fig. 1).

**Fig. 1.**
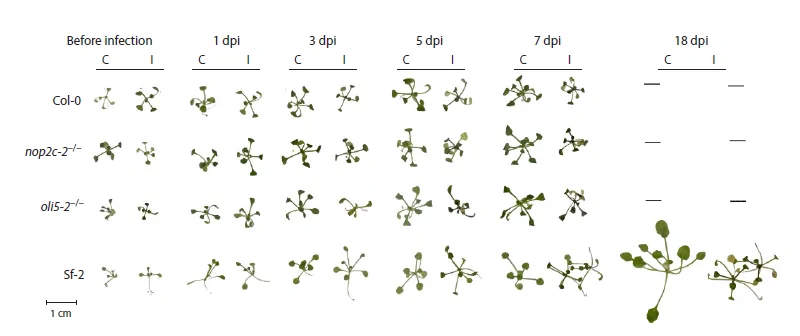
Progression of disease symptoms in A. thaliana seedlings following infection (I) and in control plants (C). Col-0, wild type; oli5-2–/– and nop2c-2–/–, mutant lines in the Col-0 genetic background; Sf-2, a long-telomere ecotype

Notably, the long-telomere ecotype Sf-2 showed the highest resistance to infection among all plant lines analyzed. Beginning at five dpi, infected plants demonstrated redu- ced growth, shown as smaller leaves comparing to the control group (Fig. 1). Symptoms of tissue damage, including chlo- rosis and leaf necrosis, were first observed after 14 dpi. The major part of shoots showed visible signs of infection only at 18 dpi.

Thus, our results indicate that different A. thaliana genotypes exhibit distinct levels of resistance and stress responses to biotic stress caused by phytopathogenic bacteria. Among the lines examined, the oli5-2–/– mutant, which displays a telomere-shortening phenotype, was the most susceptible to infection, whereas the long-telomere ecotype Sf-2 exhibited the highest level of resistance. Taken together, these observations suggest a possible association between telomere length and resistance to phytopathogenic bacteria.

**Effect of bacterial infection on telomere length in A. thaliana**

The plant infection experiment revealed that the oli5-2–/– mutant line, characterized by shortened telomeres, was the most susceptible to infection by P. syringae DC3000. More generally, plant lines with shorter telomeres tended to exhibit lower resistance to this phytopathogen. To further investigate the relationship between infection and telomere dynamics, telomere lengths were analyzed in infected and control plants. Telomere length was measured using the PCR-based PETRA (Primer Extension Telomere Repeat Amplification) assay, which enables accurate analysis of telomere length using small amounts of plant material and low DNA input (Heacock et al., 2004; Watson, Shippen, 2007). In addition, PETRA allows determination of telomere length at individual chromosome arms, facilitating the detection of statistically significant differences even when absolute changes in telomere length are relatively small.

Telomere lengths were analyzed at the short arm of chromosome 4 (4R) and at the short and long arms of chromosome 5 (5L and 5R, respectively). Measurements were performed at three days post infection, when differences in disease symptoms and stress responses among the plant lines were clearly apparent. Telomere shortening at the short arm of chromosome 4 was observed in infected plants compared to controls in all three plant lines (see the Table, Fig. 2). However, statistically significant shortening was detected only in the nop2c-2–/– mutant line, in which mean telomere length decreased from 6.14 ± 0.39 kb in control plants to 5.19 ± 0.16 kb in infected plants, with an average shortening of 0.95 kb (p = 0.0042) (see the Table, Fig. 2). In the oli5-2–/– line, which displayed the most severe visible disease symptoms at this time point, telomere shortening was also observed, with mean telomere length decreasing from 3.24 ± 0.17 kb in control plants to 3.11 ± 0.16 kb in infected plants (average shortening of 0.13 kb). However, this difference was not statistically significant (p = 0.20). A similar pattern was observed in the wild-type Col-0 line, in which telomere length decreased from 4.62 ± 0.49 kb in control plants to 4.10 ± 0.11 kb in infected plants (average shortening of 0.52 kb; p = 0.24) (see the Table, Fig. 2).

**Table 1.**
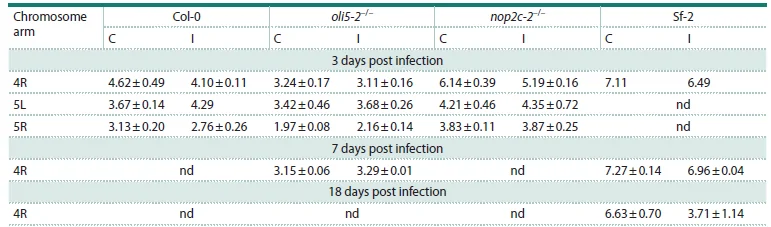
Telomere lengths of A. thaliana lines under control conditions and following infection with Pseudomonas syringae pv. tomato DC3000 Note. C – control plants; I – infected plants; nd – no data.

**Fig. 2.**
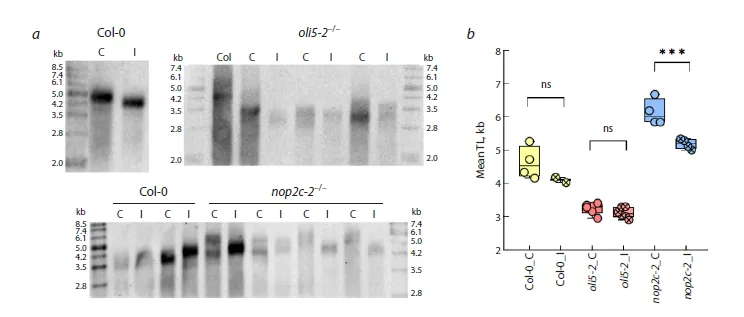
Telomere length at the short arm of chromosome 4 (4R) in Col-0, oli5-2–/–, and nop2c-2–/– plants at three days post infection. a – Southern blot analysis of PETRA products; b – mean telomere length (kb). Statistical significance was assessed using Student’s t-test. ns, not significant; *** p ≤ 0.001.

To assess telomere dynamics at later stages of infection, an additional analysis of telomere length at chromosome arm 4R was performed in oli5-2–/– plants at seven dpi. At this later stage, when disease symptoms were substantially more visible, telomeres in infected plants were significantly longer than in control plants (3.29 ± 0.01 kb vs 3.15 ± 0.06 kb, respectively; p = 0.025) (see the Table, Fig. S3).

The dynamics of telomere length changes at chromosome arm 4R in the long-telomere ecotype Sf-2 closely paralleled the progression of infection in this plant line (see the Table). At three and seven dpi, when no obvious signs of disease or growth retardation were observed, telomere length did not differ from that of control plants. In contrast, by 18 dpi, when plants displayed pronounced symptoms of biotic stress, a significant reduction in telomere length at chromosome arm 4R was detected.

To determine whether the observed changes in telomere length were common to multiple chromosome arms, we additionally analyzed telomeric loci on chromosome 5 using primers specific to long and short chromosome arms (5R and 5L, respectively). However, a controversial trend in telomere length regulation was observed at chromosome arms 5R and 5L. At the 5L arm, telomeres tended to be longer in infected plants than in the corresponding controls across all plant lines examined, although these differences were not statistically significant. In contrast, no differences in telomere length were detected at the 5R arm (see the Table). The difference between the results obtained for chromosome arm 4R and those observed at chromosome 5 may be explained by the fact that telomere length dynamics can be regulated independently at different chromosome arms within the same organism (Graakjaer et al., 2003; Perner et al., 2003; Sholes et al., 2022; Karimian et al., 2024).

Taken together, these results indicate that infection with the phytopathogen P. syringae DC3000 is associated with alterations in telomere length in A. thaliana, and that these changes depend on plant genotype.

Despite the long history of telomere research, relatively little is known about telomere length regulation in response to viral or bacterial infections and parasitic interactions. Moreover, the limited number of available studies have been conducted primarily in animals or clinical human samples. To date, reliable information regarding the effects of biotic stress on plant telomeres remains lacking. In the present study, we used infection of A. thaliana plants of different genotypes with the phytopathogenic bacteria P. syringae DC3000 as a model system to investigate changes in telomere length. Our principal finding is the identification of an association between infection and telomere length dynamics, as well as evidence that telomere length changes depend on plant genotype. Although only a limited number of natural ecotypes and mutant lines were examined, the present results lay ground for future studies of telomere regulation and chromatin stability in the context of plant disease and plant-microorganism interactions.

Evidence for an association between biotic stress and telomere length regulation is particularly well established in virus-derived diseases of humans and animals. On the one hand, viral infections can induce genomic DNA damage, telomere shortening, and telomere dysfunction. Several studies have demonstrated that chronic viral infections are associated with damage to host telomeric DNA and progressive telomere shortening (Bellon, Nicot, 2017; Dowd et al., 2017; Ji et al., 2019; Lim et al., 2022). On the other hand, viral effects on telomere biology may also be mediated through increased telomerase activity and subsequent oncogenic transformation of infected cells. Both DNA and RNA oncogenic viruses have been shown to interact with telomerase, either by integrating into host telomeric regions or by activating telomerase expression through promoter regulation (Salimi-Jeda et al., 2021). Viruses such as Epstein–Barr virus (Kamranvar, Masucci, 2017), human papillomavirus (Liu X. et al., 2008), and hepatitis B virus (Salimi-Jeda et al., 2021) can disrupt telomere and telomerase regulation, thereby contributing to cellular immortalization and tumor development. In addition, several viruses have been reported to induce alternative lengthening of telomeres (ALT) pathways (Zhu et al., 2017; Lippert et al., 2021; Tornesello et al., 2022).

Beyond studies of viral infections in humans, several investigations have examined the effects of other forms of biotic stress on telomere length in animals. For example, malaria infection has been shown to promote telomere shortening in bird species such as tawny owls and blue tits and to affect their lifespan (Karell et al., 2017; Sudyka et al., 2019). In contrast, a study of Soay sheep found no association between leukocyte telomere length and infection by parasitic nematodes, leading the authors to conclude that the costs of immune maintenance are not directly linked to telomere length in this species (Ravindran et al., 2024).

Our results indicate that plant lines with shorter telomeres were more susceptible to phytopathogenic infection and exhibited telomere shortening at earlier stages following infection. These observations raise the question of whether initial telomere length is associated with resistance to infection. Could telomere length serve as a reliable prognostic biomarker for predicting susceptibility or, conversely, enhanced resistance to biotic stress? Addressing this question will require studies involving larger and more diverse populations, including natural A. thaliana ecotypes spanning a broad range of telomere lengths, as well as additional mutants affected in known regulators of plant telomere maintenance.

To date, large-scale investigations of the relationship between telomere length and infection have been conducted primarily in the context of human viral diseases. Interest in this topic increased substantially following the COVID-19 pandemic, when several studies reported an inverse association between telomere length and the severity of clinical outcomes in hospitalized patients. These findings suggest that longer telomeres may be associated with enhanced immune function and reduced susceptibility to severe COVID-19 outcomes (Dos Santos et al., 2021; Vos et al., 2025). However, large-scale studies examining the relationship between telomere length and infectious diseases in both natural animal populations and human cohorts are complicated by numerous confounding factors, including differences in sample size, age structure, infection type and severity, telomere measurement methodology, and the tissues analyzed. Furthermore, establishing a causal relationship between telomere length and susceptibility to infection or disease severity remains challenging (Tunnicliffe et al., 2024).

Another important question raised by our findings concerns the mechanism through which phytopathogenic infection influences telomere length regulation in plants. We hypothesize that one possible mechanism involves the detrimental effects of reactive oxygen species on cellular homeostasis and chromatin stability. ROS levels are known to increase during plant immune responses to biotic stress. Under non-stress conditions, ROS produced by plant cells function as important signaling molecules involved in the regulation of numerous cellular processes, and their production is tightly controlled (Guo et al., 2023; Sood, 2025). However, during biotic stress, including phytopathogen recognition and infection, ROS production increases substantially. Elevated ROS levels contribute to several key components of plant immunity, including direct antimicrobial activity against pathogen membranes, proteins, and nucleic acids; activation of signaling pathways and immune-response cascades; and induction of genes associated with stress responses and systemic acquired resistance (SAR) (Sewelam et al., 2016; Zhang M., Zhang S., 2022; Liu C. et al., 2024; Haghpanah et al., 2025; Sood, 2025). At the same time, intracellular ROS homeostasis must remain tightly regulated, as excessive ROS accumulation can cause oxidative damage to cellular proteins, lipids, and nucleic acids, ultimately leading to programmed cell death (Vanderauwera et al., 2011; Dvorak et al., 2021; Sahu et al., 2022; Guo et al., 2023; Sood, 2025).

Telomeres may be particularly vulnerable to oxidative damage. Because telomeric DNA is enriched in guanine, the most readily oxidized of the canonical DNA bases, telomeric regions represent preferential targets of ROS-mediated damage. Oxidation of guanine residues results in the formation of lesions such as 8-oxoguanine (8-oxo-G), contributing to genomic instability (Castillo-González et al., 2022). Indeed, telomeres have been reported to accumulate disproportionately high levels of oxidative damage. Under oxidative stress conditions, telomeric DNA accounts for approximately 0.2–15 % of the total cellular 8-oxo-G content, despite representing only 0.02–0.07 % of the genome. Consequently, telomeres may accumulate 8-oxo-G at frequencies up to 100-fold higher than the remainder of the genome (Castillo-González et al., 2022). In addition, reduced telomerase activity may further exacerbate the detrimental effects of oxidative stress on telomere maintenance. Consistently with this hypothesis, decreased telomerase activity has been associated with telomere dysfunction in A. thaliana plants exposed to spaceflight conditions, a model characterized by elevated oxidative stress (Barcenilla et al., 2023).

The use of mutant lines remains an important approach for investigating telomere biology and telomere responses to environmental stress in model organisms (Campitelli et al., 2022; Castillo-González et al., 2022; Barcenilla et al., 2023). In the present study, we analyzed two A. thaliana mutant lines carrying mutations in the OLI5/RPL5A and NOP2C genes. Both gene products are known components of the ribosome biogenesis pathway (Fujikura et al., 2009; Burgess et al., 2015; Kobayashi, 2018; Abdulkina et al., 2019). Our results showed that oli5-2–/– plants were more susceptible to infection than nop2c-2–/– plants. However, significant telomere shortening at three dpi was detected only in the nop2c-2–/– line. In contrast, telomere length in oli5-2–/– plants decreased slightly at three dpi and increased at seven dpi, suggesting dynamic regulation of telomere length during the progression of stress.

The observed relationship between mutations in ribosomeassociated genes, susceptibility to infection, and telomere dynamics may reflect the central role of ribosomes in plant adaptation to environmental stress, including pathogen challenge. Under both biotic and abiotic stress conditions, protein translation represents a major regulatory target through which cells reduce energy consumption by selective synthesis of proteins involved in stress adaptation. For example, studies of plant ribosomes have revealed stress-dependent expression patterns of genes encoding proteins of both the large (RPL) and small (RPS) ribosomal subunits (Fakih, Germain, 2025). Furthermore, silencing of the ribosomal protein genes NbRPSaA, NbRPS5A, and NbRPS24A in Nicotiana benthamiana was shown to reduce the translation of defense-related transcripts, whereas silencing of NbRACK1A impaired the translation of specific antioxidant enzymes (Fakih et al., 2023). In addition, proteins involved in ribosome biogenesis, including rRNA methyltransferases, have been implicated in stress resistance. M. Schosserer et al. (2015) demonstrated that reduced expression of NSUN5-family rRNA methyltransferases, which include the NOP2C homolog, increases lifespan and stress resistance in yeast, nematodes, and flies. Notably, loss of NSUN5 alters ribosome conformation in yeast, affecting translational fidelity and promoting selective recruitment of mRNAs involved in oxidative stress responses (Schosserer et al., 2015).

## Conclusion

In summary, the present study demonstrates that infection of A. thaliana with the phytopathogenic bacterium P. syringae DC3000 is associated not only with the development of disease symptoms but also with changes in telomere length regulation. Since telomeres play a central role in maintaining genome stability, these findings suggest that telomere dynamics may represent an important component of plant responses to biotic stress. Our results further indicate that infection-associated changes in telomere length depend on plant genotype. In particular, the nop2c-2–/– mutant line exhibited significant telomere shortening at an early stage of infection, whereas the long-telomere ecotype Sf-2 showed both the highest resistance to infection and stable telomere length during the early stages of disease development. Taken together, these findings provide the first evidence of an association between phytopathogenic infection and telomere length dynamics in plants. The results establish a foundation for future studies aimed at understanding the molecular mechanisms linking biotic stress, genome stability, and telomere regulation. In the longer term, telomere length and telomere-associated pathways may have potential as biomarkers of plant stress resistance and as targets for the development of crop varieties with enhanced tolerance to pathogens and other environmental stresses.

## Conflict of interest

The authors declare no conflict of interest.
